# Integrating COPD care in Italy

**DOI:** 10.1186/2049-6958-9-26

**Published:** 2014-05-19

**Authors:** Peter MA Calverley

**Affiliations:** 1 School of Ageing and Chronic Disease, University of Liverpool, Liverpool, UK

## 

Doctors are not short of advice about how to manage their patients. In the case of COPD (Chronic Obstructive Pulmonary Disease) there are a wealth of guidelines, strategies and position papers synthesising an increasing body of data from observational studies and clinical trials into schemes that should help the doctor care for their COPD patients more effectively. In recent years particular attention has been paid to the views of the joint ERS/ATS COPD guidelines [1] and the regularly updated recommendations of the Global initiative for chronic Obstructive Lung Disease (GOLD) [2]. Now a new Italian document has been developed representing the combined efforts of three key Italian respiratory societies AIMAR, AIPO, SIMeR and the society of General Practice, SIMG, whose combined expertise covers almost all those who offer care to the COPD patient. So what does this new guidance tell us and does it add to what we have heard already?

Appropriately this new document takes a very wide view of the problems of COPD. It emphasises the need for early accurate diagnosis supported when needed but supplementary physiological investigations such as the measurement of lung volumes and airway responsiveness. It points out the limitations of relying on a fixed FEV_1_/FVC ratio of 70% or less to support the diagnosis of COPD and suggest instead that the lower limit of normal (LLN) for this ratio is a more reliable guide. There might have been a little more advice for the busy clinician on the best way of accessing appropriate LLN data and perhaps a little more caution on the usefulness of bronchodilator testing in patient evaluation [3]. However there are some excellent figures to guide diagnosis and management as well as useful tabular summaries of what tests should be undertaken by which clinicians and how to approach smoking cessation.

The authors have avoided some of the controversy and uncertainty surrounding the new GOLD severity assessment scheme [4], instead focussing on patient sub-groups that correspond to those seen in clinical practice and in clinical trials. Their treatment recommendations are not formally evidence based but closely follow those in guidelines where more extensive evidence evaluations have been conducted [5]. The authors offer clear practical guidance with support from selected but relevant literature citations which help the clinician understand why particular treatment choices have been made. There is an appropriate and pragmatic section emphasising the value of pulmonary rehabilitation together with a particularly helpful review of the role of telemedicine in COPD care, a topic scarcely touched on in other guidelines.

This new approach to COPD care is needed in Italy now. As the authors point out, COPD remains a major health problem despite much good work to increase smoking cessation rates and improve air quality. International guidelines offer an overview of COPD care in all settings but there is still a need to develop management strategies which work locally and reflect the needs and perceptions of local physicians and patients. This new initiative has achieved both of these goals admirably.
